# A Brief Review on Concurrent Training: From Laboratory to the Field

**DOI:** 10.3390/sports6040127

**Published:** 2018-10-24

**Authors:** Spyridon Methenitis

**Affiliations:** Sports Performance Laboratory, School of Physical Education & Sports Science, National and Kapodistrian University of Athens, Ethnikis Antistassis 41, Daphne, 172 37 Athens, Greece; smetheni@phed.uoa.gr; Tel.: +30-210-7276173; Fax: +30-210-7276028

**Keywords:** concurrent exercise, mammalian target of rapamycin, peroxisome proliferator-activated receptor-γ coactivator, adenosine monophosphate-activated protein kinase

## Abstract

The majority of sports rely on concurrent training (CT; e.g., the simultaneous training of strength and endurance). However, a phenomenon called “Concurrent training effect” (CTE), which is a compromise in adaptation resulting from concurrent training, appears to be mostly affected by the interference of the molecular pathways of the underlying adaptations from each type of training segments. Until now, it seems that the volume, intensity, type, frequency of endurance training, as well as the training history and background strongly affect the CTE. High volume, moderate, continuous and frequent endurance training, are thought to negatively affect the resistance training-induced adaptations, probably by inhibition of the Protein kinase B—mammalian target of rapamycin pathway activation, of the adenosine monophosphate-activated protein kinase (AMPK). In contrast, it seems that short bouts of high-intensity interval training (HIIT) or sprint interval training (SIT) minimize the negative effects of concurrent training. This is particularly the case when HIIT and SIT incorporated in cycling have even lower or even no negative effects, while they provide at least the same metabolic adaptations, probably through the peroxisome proliferator-activated receptor-γ coactivator (PGC-1a) pathway. However, significant questions about the molecular events underlying the CTE remain unanswered.

## 1. Introduction

Systematic training aims to increase the physical/fitness performance of each athlete or individual. One of the pillars of training theory is the training supercompensation cycle, firstly described by Yakovlev [1]. According to this theory, the physical loads from each training session and/or period serve as a potential stimulus which leads to a reaction of the human body [1]. Indeed, training loads induce stress in the human body, which is not familiar with the physical loads that it is forced to overcome during training. These training loads, and most importantly the training-induced stress, cause fatigue and acute reduction in both athlete’s performance and work capability (first phase of supercompensation cycle). After the end of the training session or period, an extensive fatigue is observed [1]. During this stage the body is under an emergency situation. Consequently, in the consecutive hours and days following the end of a training session or period, a pronounced recovery process is taking place, which leads to the return to the pre-training levels of physical/fitness performance and work capability. However, at this point, the body continues to react to the stimulation of the previous training session or period [1]. Thus, in this situation, the work capability and the physical/fitness performance of each athlete continues to increase, causing improvements in performance, the supercompemsation phase [1]. From the time that Yakovlev introduced the theory of the training supercompensation cycle until now, scientists have provided strong evidence that the training-induced adaptations occur through a complex network of several molecular pathways that are specifically stimulated during training. Sports and daily related activities are commonly classified as either “endurance-based” or “strength–power-based”. Numerous studies investigate the one-dimensional demands underlying each type of activity and provide strong evidence about the specific adaptations from the uncomplicated stimulus, according to the principle of training specificity. Indeed, endurance training stimulates molecular pathways [like those of PGC-1, Ca^2+^/calmodulin-dependent kinases (CaMK), calcineurin, adenosine monophosphate-activated protein kinase (AMPK) and mitogen-activated protein kinases (ERK1/2, p38 MAPK)] underlying the cellular processes that promote mitochondrial protein synthesis–biogenesis and angiogenesis, and thus providing metabolic adaptations leading to an increase in endurance capacity [2]. In contrast, resistance training promotes mostly the increase of muscle hypertrophy, strength and power via the AKT-mTOR (mammalian target of rapamycin) pathway that stimulates myofibrillar protein synthesis.

## 2. The Concurrent Training Effect

In contrast to the experimental settings, in the “real” world and for the general population, the combination of both resistance and endurance exercise in a training program leads to superior adaptations in health-related and body function variables, independent of age or sex, including increases and/or improvements of basal metabolic rates, insulin sensitivity, glucose/lipids metabolism, lipidemic profile and body composition, while both muscular hypertrophy/strength/power and endurance capacities are increased [3,4,5]. Additionally, the majority of sports are neither “endurance-based” nor “strength–power-based”, but of a mixed type, with performance to be determined by the specific contribution of muscle strength/power and endurance, which varies between sports. In addition, even in long-distance runners or cyclists, strength training routines result in a significant increase of their endurance performance [6]. Thus, coaches aim to maximize training adaptations from both training modules by what is known as “concurrent training” (e.g., simultaneously training for both strength and endurance regiment). 

Hickson, with his pioneering work in early 1980s, reported that after a concurrent training (CT; e.g., the simultaneous training of strength and endurance) intervention, resistance training-induced adaptions were lower compared to those that occurred when individuals perform only resistance training [7]. Hickson referred to this phenomenon as the “Interference effect”, however, it is now known as the “Concurrent training effect” (CTE). Until now, many studies have investigated the effect of CT programs on both resistance and endurance training adaptations, with many controversial results. The majority of them provide strong evidence that after CT intervention muscle hypertrophy, strength and power adaptations were mostly attenuated, compared with those after isolated strength training stimuli [8,9,10,11,12,13,14,15,16,17]. By contrast, there are several studies providing strong evidence that resistance training adaptations are not suppressed, but further increased after CT [15,18,19,20,21,22,23,24,25]. It seems that CT innervations do not affect negatively the endurance training adaptations [16,17]. Furthermore, it seems that after CT, athletes’ endurance capacity is increased to a greater extent compared to when endurance training is performed alone [6,25,26,27,28,29,30]. However, recently it has been suggested that, maybe, longitudinal CT programs may impair the endurance training adaptations in well-trained runners [31]. 

Considering these controversial results, it seems that some individuals/athletes experience strength adaptations that are negatively affected after CT, while others experience significant gains with CT [32]. Unfortunately, the reasons for the differential responses/adaptations between participants after CT have not been fully investigated and there are still many unanswered questions. CTE is a multidimensional phenomenon, affected by various physiological and non-physiological factors, such as the exercise characteristics, training background, muscle groups that are trained, inter-individual variations, etc. In addition, the complexity of the molecular mechanisms of the concurrent training effect make it difficult to assess. According to recent studies, it appears that CTE is mostly affected by the interference of molecular pathways underlying adaptations from each type of training segment [2,33,34,35,36,37], and the extent of muscle damage [8,35]. According to the above, it seems that an endurance training prescription can determine the magnitude of the CTE [2,8,19,33,36,37], probably in a volume-intensity-type-frequency dependent manner [8,36]. This brief review examines our current knowledge of the molecular events underlying the CTE, by summarizing mostly studies performed in humans, metanalyses and previous reviews, while occasionally studies on animals are also included when necessary. The purpose of the present brief review is not to present and/or discuss extensively the interaction of the molecular pathways after a CT, nor to discuss analytically the results of each study, but to simply and briefly explain to coaches, trainers, athletes and sports scientists the physiological basis of CT, providing them with evidence-based guidelines to minimize the negative effects of CT through manipulating the characteristics of endurance training. For the purpose of the present review, articles were obtained from PubMed, Google Scholar and MEDLINE, using the keywords “Concurrent training”, “Concurrent Exercise”, “Concurrent Effect”, “Molecular”, “Molecular Responses”, “Molecular Biology”, “Protein Synthesis Rate” “Interference”, “Combined Aerobic/Endurance and Resistance Training”, published until 2018. An article was included in the present review if it was related to the above topics. Literature that was cited in each article was also searched if needed.

## 3. The Role of Volume, Intensity and Type of Endurance Training

**Figure 1:**
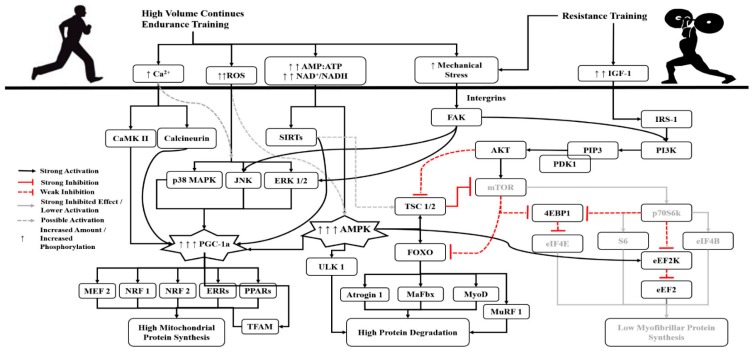
Molecular events after a concurrent training incorporating resistance exercise and high-volume, low-moderate intensity endurance exercise leads to a high increase of AMPK phosphorylation, leading to inhibited myofibrillar protein synthesis, via the de-activation of the mTOR cascade through the AMPK-mediated phosphorylation of TSC 1/2, but also to increased mitochondrial protein synthesis, via the AMPK, ROS, CaMKII, Calcineurin, Sirt1 and p38 MAPK activation of *PGC-1a*. In addition, the increased phosphorylation of AMPK mediates the increase of protein degradation, by activating FOXO, MaFbx, MuRF 1, ULK-1, MyoD and Atrogin-1 molecules. 4EBP1: Eukaryotic initiation factor 4E-binding protein 1; AKT: Protein kinase B; AMP:ATP: *Adenosine monophosphate*: *Adenosine triphosphate ratio*; AMPK: Adenosine monophosphate-activated protein kinase; Ca^2+^: Calcium ions; CaMK II: Ca^2+^/calmodulin-dependent kinases II; eEF2: Eukaryotic elongation factor 2; eEF2K: Eukaryotic elongation factor-2 kinase; eIF4B: Eukaryotic translation initiation factor 4B; eIF4E: Eukaryotic translation initiation factor 4E; ERK 1/2: extracellular signal–regulated kinases ½; ERRs: Estrogen receptor related receptor (ERR) family; FAK: Focal adhesion kinase; FOXO: Forkhead box O (*FoxO*) transcription factors; *IGF-1*: Insulin-like growth factor 1; IRS-1: Insulin receptor substrate 1; JNK: Jun amino-terminal kinase; MaFbx: Muscle-atrophy f-box; MEF 2: myocyte enhancer factor-2; mTOR: mammalian target of rapamycin (in this text we are referring to Raptor mTOR); MuRF 1: Muscle ring-finger 1; MyoD: Myoblast determination protein; NRF 1: Nuclear respiratory factor 1; NRF 2: Nuclear factor (erythroid-derived 2)-like 2 (also known as NFE2L2); p38 MAPK: p38 mitogen-activated protein kinase; p70S6k: Ribosomal protein S6 kinase beta-1 (also known as S6K1); PDK1: 3-phosphoinositide-dependent protein kinase-1; PGC-1a: peroxisome proliferator-activated receptor-γ coactivator 1a; PI3K: phosphatidylinositol 3-kinase/Phosphatidylinositol-4,5-bisphosphate 3-kinase; PIP3: Phosphatidylinositol (3,4,5)-trisphosphate; PPARs: Seroxisome proliferator–activated receptor family; ROS: Reactive Oxygen Species; S6: Ribosomal s6 kinase (also known as rsk and p70^rsk^); SIRT: Sirtuin; TFAM: Mitochondrial transcription factor A; TSC 1/2: Tuberous sclerosis complex ½; ULK 1: Unc-51-like kinase.

A significant inhibitor of AKT-mTOR pathway activation, and thus of muscle hypertrophy, is AMPK [2,33,34,35,36], which is a key energy sensor of the cells [38,39], as well as a significant regulator of mitochondrial protein synthesis–biogenesis. This probably occurs by the activation of its downstream target, peroxisome proliferator-activated receptor-γ coactivator (PGC-1a; [40,41]). Low energy availability, increased energy deficit and/or an increase of Adenosine monophosphate/ triphosphate (AMP/ATP) ratio in the cells, results in AMPK phosphorylation, and thus, in increased activation of tuberous sclerosis complex 1/2 (TSC1/2) which in turn causes the inhibition of mTOR signaling, probably by the de-phosphorylation of Raptor-mTOR [2,33,34,35,36,38,39,40]. Increases in AMPK phosphorylation, during and after a continuous endurance training seems to have a dose–response relationship with the endurance training load/volume and/or intensity, e.g., higher loads, longer durations and higher intensities resulting in higher increases of AMP/ATP ratio and thus higher activation of AMPK [24,33,42,43,44,45,46]. In addition, the increase of AMPK phosphorylation promotes the protein breakdown mechanisms by activating the Ubiquitin-proteasome and autophagy/lysosomal systems, via the activation of forkhead-box O3a (FOX-O3a), muscle-atrophy f-box (MaFbx), muscle ring-finger 1 (MuRF-1), Unc-51-like kinase 1 (ULK-1) molecules, etc. [34,35,36,46]. Indeed, according to a recent review, performing long-duration or high-volume, moderate intensity endurance exercise, in a CT, results in the inhibition of myofibrillar, but also of mitochondrial protein synthesis [46]. However, it must be noted that exercise-induced increase of AMPK signaling is not the sole moderator of the interference between the molecular cascades, but probably one of the most important [2,19,33,35,36,37,47,48,49]. Therefore, a training regimen with high energy demands will probably induce significant activation of the above mechanisms, resulting in a significant inhibition of muscle myofibrillar protein synthesis regulator pathways. Thus, high volume/distance, long-term (>20 min), moderate intensity (<85% of maximum heart rate), continuous type aerobic exercises seem to increase the CTE during a CT [8,16] (Figure 1).

**Figure 2:**
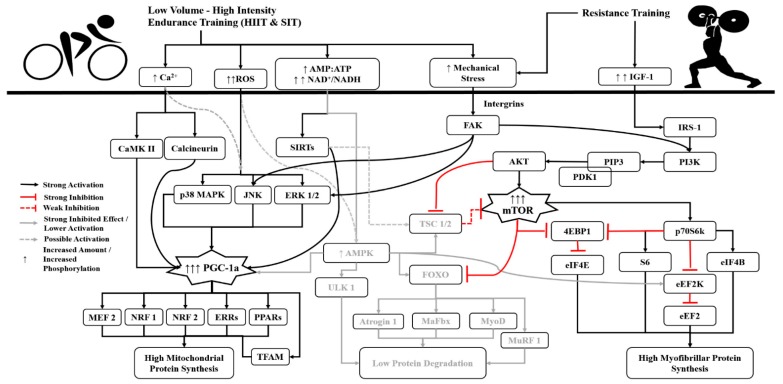
Molecular events after a concurrent training including low-volume high-intensity interval training (HIIT) or sprint interval training (SIT). A concurrent training incorporating resistance exercise, low-volume, high-intensity endurance exercise leads to a significant lower activation of AMPK, and thus to a very low activation of TSC 1/2, FOXO, MaFbx, MuRF 1, ULK-1, MyoD and Atrogin-1 molecules, allowing the increased myofibrillar and mitochondrial protein synthesis, and keeping low the rates of proteins degradation. 4EBP1: Eukaryotic initiation factor 4E-binding protein 1; AKT: Protein kinase B; AMP:ATP: *Adenosine monophosphate*: *Adenosine triphosphate ratio*; AMPK: Adenosine monophosphate-activated protein kinase; Ca^2+^: Calcium ions; CaMK II: Ca^2+^/calmodulin-dependent kinases II; eEF2: Eukaryotic elongation factor 2; eEF2K: Eukaryotic elongation factor-2 kinase; eIF4B: Eukaryotic translation initiation factor 4B; eIF4E: Eukaryotic translation initiation factor 4E; ERK 1/2: extracellular signal–regulated kinases ½; ERRs: Estrogen receptor related receptor (ERR) family; FAK: Focal adhesion kinase; FOXO: Forkhead box O (*FoxO*) transcription factors; *IGF-1*: Insulin-like growth factor 1; IRS-1: Insulin receptor substrate 1; JNK: Jun amino-terminal kinase; MaFbx: Muscle-atrophy f-box; MEF 2: myocyte enhancer factor-2; mTOR: mammalian target of rapamycin (in this text we are referring to Raptor mTOR); MuRF 1: Muscle ring-finger 1; MyoD: Myoblast determination protein; NRF 1: Nuclear respiratory factor 1; NRF 2: Nuclear factor (erythroid-derived 2)-like 2 (also known as NFE2L2); p38 MAPK: p38 mitogen-activated protein kinase; p70S6k: Ribosomal protein S6 kinase beta-1 (also known as S6K1); PDK1: 3-phosphoinositide-dependent protein kinase-1; PGC-1a: peroxisome proliferator-activated receptor-γ coactivator 1a; PI3K: phosphatidylinositol 3-kinase/Phosphatidylinositol-4,5-bisphosphate 3-kinase; PIP3: Phosphatidylinositol (3,4,5)-trisphosphate; PPARs: Seroxisome proliferator–activated receptor family; ROS: Reactive Oxygen Species; S6: Ribosomal s6 kinase (also known as rsk and p70^rsk^); SIRT: Sirtuin; TFAM: Mitochondrial transcription factor A; TSC 1/2: Tuberous sclerosis complex ½; ULK 1: Unc-51-like kinase.

In contrast, it seems that low-volume (1–2 km per training session), short bouts (4–10 min) of high-intensity interval training (HIIT) or sprint interval training (SIT) [50,51], especially when they incorporate cycling, have lower negative effect on resistance training induced adaptations through a concurrent program [8,17,34,36]. Low-volume HIIT or SIT training, seems to have lower energy demand compared with a matched volume, continuous type endurance exercise [50,51,52,53,54,55,56,57,58,59,60]. Thus AMPK and TSC1/2 activations are lower or the possible increase of AMPK is transient, returning to pre-training level nearly 3 h after the training [44,50,52,59,61,62,63]. In addition, after HIIT or SIT training, PGC-1 activation is increased to a greater extent than after work-matched long-duration endurance training [52,54,59,62,63,64], leading to mitochondrial biogenesis [41,65,66]. PGC-1a (Figure 2), has multiple roles on muscle mitochondrial function, including the upregulation of pyruvate dehydrogenase kinase (PDK) 4 and of pyruvate dehydrogenase complex (PDCP), resulting in the increase of fat oxidation in skeletal muscle cells [41]. In addition, exercise-induced PGC-1a activation from reactive oxygen species (ROS), CaMK, Calineuurin, sirtuin 1, (Sirt1) and p38 MAPK, leads to an increase in both PGC-1a gene expression and PGC-1a-mediated upregulated gene encoding of the mitochondrial metabolic proteins and transcription factor A (TFAM), which in turn translocated into the mitochondria, affecting the mitochondrial protein synthesis [41,66]. It seems that after CT incorporating resistance exercise with HIIT, PGC-1a and mTOR phosphorylation are in fact increased over a greater extent than after a single bout of resistance exercise [64]. In addition, after the combination of resistance exercise and HIIT endurance exercise, the phosphorylation of mTOR and p70S6k remains elevated, while the activation of the TSC1/2 complex remains unchanged up to 3 h post-training compared with CT incorporating resistance exercise and moderate-intensity continuous endurance exercise [44]. Finally, CT including resistance exercise and HIIT seems to inhibit (but not always) to a greater extent the proteolytic markers that exacerbate the rate of protein degradation (like MuRF-1, Atrogin-1, Myostatin, Fox-O etc.) in contrast to when continuous endurance exercise is included in a CT allowing the greater increase of myofibrillar and mitochondrial protein synthesis [44,64].

These differential molecular pathway responses, between CT incorporating either low-volume HIIT/SIT or continuous type, high-volume endurance training, have also been verified in longitudinal training studies. It seems that low volume, maximum and supramaximal HIIT/SIT [≥85% of VO_2max_ velocity (vVO_2max_) or peak power (W_peak_)] endurance training, during CT, did not result in decrements of strength, power and muscle hypertrophy adaptations, and in many cases they induce greater resistance training adaptations, while significantly increasing endurance capacity and performance, compared with high-volume low-moderate intensity endurance training [3,8,12,13,14,15,16,17,18,19,20,21,22,23,24,25,26,28,45,61,67,68,69,70,71,72,73,74,75,76,77,78]. In addition, as described above, at the molecular level it seems that when cycling is used, compared with running, there are even lower or no negative results on resistance exercise-induced adaptations, especially on strength and power performances [8,18,28,72,73,79,80,81], indicating that cycling is superior to running [82]. Thus, according to the existing literature, inclusion of high intensity (maximal or supramaximal; ≥100% of vVO_2max_ or W_peak_), low-volume (<20 min) HIIT and SIT endurance training seems to minimize the negative CTE, especially when cycling is preferred instead of running.

## 4. The Role of Training Frequency and Intra-Session Exercise Sequence

It is possible that CTE could be affected by the intra-session exercise sequence. Unfortunately, studies investigating the effect of intra-session exercise sequence, at the molecular level, are very few with controversial results. Some data indicate that acute cell-signaling responses are similar, either performing first resistance or endurance exercises in a training session [83]. In contrast, other studies support that the sequence of the training regimen has strong impact on acute molecular responses [42,84]. The same controversial results are observed in training studies. Many studies report that when endurance training is performed prior to resistance, muscle hypertrophy, strength and power performances adaptations are compromised [14,46,70,74,79,80,81,85,86,87,88,89,90,91]. Probably, by performing endurance exercises prior to resistance training, an inhibition of performance during resistance training is observed, which may lead to compromised acute molecular responses, and thus to lower resistance training-induced adaptations [14,46,74,85,86,87,88,89,92]. In contrast, several other studies suggest that intra-session exercise sequence does not compromise endurance and/or resistance training-induced adaptations after a CT program [31,86,88,89,92,93,94,95], while in some cases, the endurance–strength order may lead to greater resistance-induced adaptations on strength and power performances [96]. However, in the majority of the above short-termed CT protocols indicating that exercise sequence is not important for the extent of training adaptations when strength training was performed prior to endurance, higher but not significant resistance training-induced adaptations were observed [31,86,88,89,92]. It is possible that these non-significant higher adaptations, may have a huge impact on training-induced adaptation after longitudinal training periods (>2 years). Unfortunately, this is something that could not be answered from the existing literature, due to the fact that all the previously mentioned studies are short-term. In addition, for these controversial results, many physiological and non-physiological parameters (including sex, age, training history, training background, muscle groups that are trained etc.), have been suggested for their possible impact on CTE. However, these parameters have not been fully investigated until now, and many questions about their effect remain unanswered. Form the limited studies in this topic, it seems that the training sequence is more important to women than to men. Indeed, intra-session exercise sequence did not affect the adaptations in novice men after a CT program [86,92]. In contrast, when resistance training performed prior to endurance, women tend to have higher resistance training-induced adaptations, compared with when endurance training was performed prior to resistance, probably due the higher neuromuscular fatigue that is observed in women after endurance training [86,92]. According to the above controversial results, it is difficult to conclude which intra-session exercise sequence is the optimal for the highest CT-induced adaptations. It is possible that the most essential factor for the optimal intra-session exercise sequence is the importance of each CT regimen in the final outcome. According to the priority training principle, exercises aiming to improve the most important determinant parameters of performance should be performed firstly [1]. 

Lately, an increased number of studies have provided strong evidence that performing separated bouts of endurance and strength trainings may result in higher adaptations in endurance capacity, muscle hypertrophy, strength and power performances. Indeed, from the pioneering work of Sale et al. [97], it has been reported that performing endurance and strength training in different days and/or training sessions, may be more beneficial for higher adaptations, for both training parts of a CT period. This pioneering work has been supported by an increased number of recent studies indicating that performing in separate bouts and/or days, the strength and endurance modules, is preferred compared with one single training session that incorporates both endurance and strength training [3,98,99]. Indeed, at least in athletes, in several studies, reviews and meta-analyses, it has been revealed that for maximizing training adaptations of each training regimen in a CT, the two exercise bouts should be separated by 3–6 (if the training goal is to maximize the training adaptations of resistance exercise) or 24 h (for maximizing the training adaptations of endurance training) [8,31,36,42,46,74,84,87,89,98,99,100]. Probably, by performing in separate training bouts the two regimens of CT may result in avoidance of any possible overlap of peak activations of myofibrillar and mitochondrial protein synthesis molecular cascades, leading to simultaneous higher adaptations from both regimen [101]. However, the needed time interval between the two bouts depends on the training induced fatigue that has been caused from the first training bout, e.g., higher training loads induce higher fatigue and thus longer time intervals between the two regimens is needed [3,92,98,99,102]. For example, if endurance training is performed prior to resistance exercise, a longer time interval between the two training sessions (probably >48 h) is needed [92,102], especially in women due to the higher training-induced fatigue [86].

Taking this one step forward, in extreme cases where the resistance training adaptations are of high importance, or the training period is very limited, it seems that the frequency of endurance training may further affect the training outcomes [46,68,74]. It has been suggested that a training frequency ratio between 2:1 and 3:1 (frequency of resistance training per week: frequency of endurance training per week), leads to higher resistance training-induced adaptations, compared with when a ratio of 1:1 was performed [43,68]. According to the above, separated training sessions, and low frequency and volume of endurance training should be performed, if the primary outcomes of the training intervention are muscle hypertrophy, strength and power [8,33,34,35,36,43,45,46,68,74], in an effort to achieve a prevailing resistance exercise stimulus [48].

Taking the above together, and considering the limitations of each study and their controversial results, it could be concluded that when the aim of the training is to maximize adaptations of muscle mass—strength—power, as well as to improve body composition, resistance exercises should be performed prior to endurance exercises, and vice versa when increases in endurance capacity are necessary or when the resistance training adaptations are of lower importance [14,46,74,85,86,87,88,89,92]. However, for maximizing the training-induced adaptations from both the endurance and resistance training regimen of CT, separation of the training bouts seems to be needed, when this can be performed, while coaches should be careful with the training-induced fatigue from each bout, allowing their athletes to have at least 6–24 h rest interval between the two bouts. Finally, depending of the training goal, a ratio of 2:1 or 3:1, favoring the most crucial training regimen in each period, should be kept.

## 5. The Problem of the Acute Studies and the Role of Training History Background

Lately an increased number of studies have indicated that the training history and the years of systematic training are also significant effectors of the CTE [33]. In novice participants, several studies and reviews suggest a blunting of acute responses of the anabolic signaling promoting the myofibrillar protein synthesis after a CT [2,33,34,35,36,38,39,40,46,69]. In contrast, other studies, again in novice participants, provide strong evidence about similar acute molecular responses, when they compared the effect of a CT versus a single mode of exercise [18,44,47,49,81,103,104]. However, there is a clear CTE after a long-term interventions [8,19,33,34,35,36,46,74]. Concerning these controversial results, it has been recently suggested that the training experience and the inter-individual responses are the most important factors [19,33,46], even if there is strong evidence that individual gene responses could not explain the chronic training induced physiological and muscle morphological changes [105]. Compared with trained participants or athletes, untrained or moderate trained individuals tend to have higher activations of all molecular machineries that lead to both myofibrillar and mitochondrial protein synthesis, probably because any stimuli may induce significant perturbation to cellular homeostasis and, thus, higher training-induced adaptations are observed [33,106,107,108]. Indeed, in novice/untrained individuals, even after a single bout of endurance exercise stimuli, there is a significant upregulation of mTOR cascade [109,110,111,112,113,114,115] leading to modest gains in muscle size/strength/power [116], while after a resistance training session the molecular pathways promoting mitochondrial protein synthesis are also increased [115,117,118] and thus, oxidative capacity is increased [78]. In addition, when experienced resistance or endurance trained individuals performed a single bout of their non-familiar training stimuli, there was a cross-talk phenomenon [100]. The activation of the molecules that control mitochondrial protein synthesis was higher when resistance-trained individuals performed endurance exercise compared with endurance trained participants, while the phosphorylation of the mTOR cascade proteins was higher when endurance-trained participants performed resistance training compared with resistance-trained [100]. These results provide strong evidence that the training history of well-trained participants has a strong impact on the acute, specific signaling responses after divergent exercise stimuli, especially when the new stimuli is at the end of the endurance–resistance training continuum [33,100]. According to the above, it is not surprising that in novice/recreational participants, sοme studies provide evidence that there is a greater increase of all molecular pathways controlling both myofibrillar and mitochondrial protein synthesis after a CT, leading to significant adaptations in both regimens, compared with a single bout of either resistance or endurance exercise [18,44,47,49,81,103,104]. However, long-term exercise interventions (a significant limitation for our understanding of the CTE is that the training interventions ranged between 5–20 weeks only) tend to blunt the possible overall greater increases of anabolic responses after the initial concurrent exercise stimuli [18,33,35,37]. Probably, as the months or the years of the training are increased, and the training loads of each divergent exercise are also increased to disrupt homeostasis, the repeated bouts of both endurance and resistance training regimens of a CT may generate specific training adaptations, resulting in muscle phenotype transformations and/or in specific muscle damage protection mechanisms by blunting the molecular responses after each training session [33,36]. These alterations may lead to the impaired resistance exercise-induced adaptations after a CT, in well trained individuals [33,36]. These adaptations or proposed mechanism may explain the observed significant reduced adaptations on neuromuscular performance, after CT in well-trained participants; however, so far they have never been investigated. According to the above it seems that CTE is stronger to well-trained individuals, who repeatedly receive the same training stimuli, than in novices [33]. However, the exact mechanisms, or when these proposed specific adaptations start to impair the adaptations through a CT, or what manipulations of the training plans should be done to avoid it, are still under investigation, and there is not enough evidence for safe and reliable conclusions. 

## 6. Conclusions

In conclusion, and according to the results and the limitations of the present literature investigating the CTE, it seems that high-volume, moderate, continuous and frequent endurance training, are thought to negatively affect the resistance training-induced adaptations, probably by inhibition of AKT-mTOR pathway activation, from the adenosine monophosphate-activated protein kinase (AMPK). In contrast, it seems that low-volume, short bouts of HIIT or SIT, especially when they incorporate cycling, have lower or even no negative effect on resistance training-induced adaptations through a concurrent program, while they provide at least the same metabolic adaptations; probably through the peroxisome proliferator-activated receptor-γ coactivator (PGC-1a) pathway. Thus, sports scientists but mostly the coaches should be very careful about the training plans/strategies which they are willing to follow, when a combination of endurance and resistance training is needed, by taking into consideration how different training modalities interact between them. In an effort to maximize the training adaptations from a CT and thus limit the CTE, sports scientists and coaches should consider the following suggestions:

(1) The level of fatigue from both modules and the need of inter-stimulus or inter-session time intervals to minimize the training induced overall fatigue.
(2) Consider the training volume of each training regimen of a CT, in an effort to minimize muscle fatigue and energy expenditure.
(3) Incorporate low-volume, high-intensity (maximum and supra-maximum) HIIT or SIT endurance exercises, in an effort to keep low the activation of AMPK.
(4) Where possible, prefer cycling over other types of endurance training.
(5) When the goal is to maximize the resistance training adaptations on muscle mass—strength—power, as well as improve body composition, resistance exercises should be performed prior to endurance exercises.
(6) When the goal of the training is to increase the endurance capacity or when the resistance training adaptations are of lower importance, then endurance exercises should be performed prior to resistance exercises.
(7) Separating training bouts by 3–6 to 24 h, even if this is not always practical to the “real” world of athletes’ training.
(8) Strong consideration of the frequency of each training stimulus. If resistance training-induced adaptations are of importance, consider using a ratio of 2:1 or 3:1 between resistance training sessions per week: endurance training sessions per week. In contrast, it seems that a ratio of 1:1 or 1:2 leads to a better improvement of endurance capacity.
(9) Training experience and background are of high importance for the CTE, which is stronger for experienced participants, while in novice or recreational individuals it is lower. Thus, training plans aiming to maximize performance in well-trained individuals or athletes, through a CT intervention, should be designed very carefully, based on the specific requirement of each sport as well as on the evidence-based suggestions as described above. However, a “new” training stimulus in well-trained individuals, should lead to high and very quick adaptations. Thus, keeping low the volume and the frequency but high the intensity of the new training stimulus may result in increased adaptations from the new training regimen, without, at least in theory, limiting the progression of the commonly used stimuli.

## 7. Questions to Be Answered in Future Studies

Until now our knowledge about the molecular events that underly CTE has been limited. Future studies must be longitudinal, with the training interventions to exceed three months, and should aim to answer the following questions:

(1) What is the meaning of the acute molecular events that are present after the initial training session for the training-induced adaptations after a longitudinal training intervention?
(2) How is the time-course change of molecular mechanisms responding?
(3) Where is the critical time-point of a training intervention after which the CTE is stronger?
(4) What is the meaning of intra-individuals’ molecular mechanism responses during a CT?
(5) Is there a dose-response relationship between volume—intensity—type—frequency of both endurance and resistance exercise during a CT?
(6) What are the characteristics of the responders and non-responders?
(7) What are the effects of CT, including power and endurance training?
(8) What are the effects of CT in well-trained endurance and/or resistance-trained individuals and/or athletes?
